# Effects of coaches’ feedback on psychological outcomes in youth football: an intervention study

**DOI:** 10.3389/fspor.2025.1527543

**Published:** 2025-02-27

**Authors:** Iben Berntzen, Pål Lagestad

**Affiliations:** Faculty of Teacher Education and Arts, Nord University, Levanger, Norway

**Keywords:** soccer, experience, being seen, development, joy, motivation, well-being, achievement

## Abstract

**Introduction:**

The purpose of this study was to examine how feedback from the coach influences football players' experiences of well-being, mastery, pleasure, satisfaction, development, being seen by the coach, and motivation in two different groups.

**Methods:**

The study used an intervention with a crossover experimental approach, where 95 players aged 14–18 that participated in sports clubs and specialized football classes in a town in Mid-Norway, participated in the same organized training session with the same coach in every session—one without feedback and one with feedback from the coach, answering a questionnaire after each training session. The questionnaire was developed with a total of seven questions with high face validity for each of the variables. The study was approved by the Norwegian data protection agency, and a written informed consent for participation in this study was provided by the participants' legal guardians/next of kin. Wilcoxon nonparametric tests were used to examine differences between the session without and the session with feedback among players in the two groups for each variable.

**Results:**

Analyzing all players, the results showed that when feedback was given, it led to a significant increase in mastery, the experience of being seen by the coach, and motivation, compared to the same training session when they did not receive feedback. Furthermore, analyzing only players who participated in sports studies with a specialization in football, the results showed that giving feedback led to a significant increase in well-being, pleasure, satisfaction, and development. Finally, analyzing only players who participated in sports teams and did not specialize in football in sports programs showed that when feedback was given, it led to a significant increase in, the experience of being noticed by the coach, and motivation—but a decrease in development, compared to the same training session when they did not receive feedback.

**Discussion:**

This study introduces a unique and novel intervention approach focusing on the effects of feedback. The findings suggest that football coaches' feedback has a positive outcome for several psychological factors of young players. The findings of the study highlight the importance of football coaches' feedback.

## Introduction

Previous empirical findings regarding the psychological impact of feedback indicate a positive effect of feedback. According to Hattie and Timperley, feedback is one of the methods with the strongest impact on skill development (1) and is among the most effective ways to promote learning and development (1, 2). To ensure that each student experiences good teaching and visible learning, the amount and quality of feedback from the teacher are crucial for future learning outcomes (2). Hattie (2) and Hattie and Timperley (1) argue that the most important factor for learning lies in daily feedback, that is, immediate feedback. This means feedback given through dialogue or constructive messages. Even if these theories are aimed at school and education, we argue that both teachers and coaches work towards the same main aim—learning and development, and that theory and research related to feedback in an educational setting are also applicable in sport settings. Olympiatoppen is an organization in Norway responsible for the development and support of elite sports. Olympiatoppen's main role is to provide resources, training, and support to Norwegian athletes to help them achieve success in international competitions According to Olympiatoppen (3), feedback should be used to reinforce something positive. This study will address feedback and how it affects young football playerś experiences of well-being, mastery, pleasure, satisfaction, development, being seen by the coach, and motivation.

The significance of feedback in this study is based on Hattie (2) and Hattie and Timperley (1), who emphasize the importance of feedback for development, but also on the pedagogical term “being seen” identified in physical education (4, 5), which we argue is also important among young athletes. In an interview study of 26 students, four factors were identified that contribute to students’ experience of being seen, with the quality of feedback being one of these (4). The four factors in the study were: that the youths can showcase their skills, that the leader of the youths shows care, the quality of feedback from the leader, and the quality of the dialogue between the leader and the youths themselves. From a theoretical point of view, the importance of feedback can be explained by recognition theory, where the players feel their competencies are being valued and recognized through feedback (6), and that coaches’ feedback is a strategy that involves giving praise and appreciation, and acknowledgment of what the person does. It can also be explained by self-determination theory (7) where the feeling of competence, autonomy and social belonging are fundamental needs.

With a quantitative approach, Andresen et al. (5) found that the same factors were significant and highly correlated. According to Hattie and Timperley (1), feedback is extremely important for development. The purpose of feedback is to reduce the gap between the student's current understanding and the desired goal. Hattie and Timperley further argue that the most critical factor for learning occurs through daily feedback given during the development process, specifically through immediate feedback. This refers to feedback given through dialogue or constructive messages. The key questions here are: “Where is the player, where should the player go, and how does the player get there?”

According to Schmidt and Lee (8), feedback consists of two different categories: intrinsic and augmented feedback. Intrinsic feedback refers to the perceptual-cognitive information perceived through the senses (e.g., sight, hearing, touch) before, during, or after an action. Augmented feedback can be defined as information that supplements intrinsic feedback. In this study, feedback will mainly be augmented feedback.

There are various methods for providing feedback, and according to Hattie (2), feedback can be given through increased effort, motivation, affective processes, and engagement. The goal of feedback is to reduce the gap between students’ current understanding, effort, and learning goals. As highlighted by Hattie (2), three conditions must be present for feedback to be useful and effective. The student must need the feedback, be willing to use it, and the feedback must be given at the right time.

This is supported by other studies (9, 10).

Gökçe (11) found that positive feedback contributed to increasing youths’ perceived mastery and goal orientation regarding skill acquisition. Feedback plays an important role in shaping students’ performance goals and perceived motivational climate. Therefore, teachers should be aware of how they provide feedback to promote the development and mastery of skills. Additionally, a study by Buchanan and Wang (12) show that feedback can have a positive impact on skill development.

In a systematic review and meta-analysis aimed at establishing the evidence for the effects of feedback on acute resistance training performance and chronic training adaptations, Weakley et al. (13) demonstrated a positive influence of feedback, with all outcomes showing superior results compared to when no feedback was provided. A literature search shows that there is considerable previous research on the importance of feedback for development and learning, but few interventions have been conducted regarding the psychological impact of feedback in sports and how feedback affects development, well-being, motivation, and mastery. A study by Robin et al. (14) found that verbal feedback has a positive effect on the accuracy of passes among football players. It has also been argued that individual, concrete, and reflective feedback is preferable for skill development (15). Additionally, positive feedback has been found to have a positive effect on both motivation and skill development (16). This aligns with the study by Smith and St. Pierre (17), where 85% of participants indicated that the teacher had a significant impact on their experience of physical education. A key factor for student well-being was reported to be the interaction between teacher and student. This included the teacher's ability to communicate effectively with students, encourage them, and provide positive feedback, and it can be argued that this also applies to coaches and athletes.

Previous empirical findings regarding the psychological impact of feedback presented in the discussion above refer to some research on the effect of feedback, but no one has previously looked at the significance of feedback for young football players through an intervention study. We argue that feedback may affect well-being, mastery, pleasure, satisfaction, development, being seen by the coach, and motivation positively. In this study the definitions of the included psychological variables are created from our own experiences but also checked by AI. **Well-being**: The overall physical, mental, and emotional health of young players, ensuring they feel good and function well both on and off the field. **Mastery**: The sense of competence and skill development in football, where young players feel they are improving and mastering new techniques and strategies. **Pleasure**: The enjoyment and fun that young players experience while playing football, which keeps them engaged and enthusiastic about the sport. **Satisfaction**: The feeling of contentment and fulfillment that young players get from achieving their goals, whether it's winning a game, improving their skills, or simply enjoying the game. **Development**: The continuous growth and progress of young players in their physical abilities, technical skills, tactical understanding, and personal attributes. **Being seen by the coach**: The recognition and acknowledgment from the coach, which makes young players feel valued and appreciated for their efforts and contributions. **Motivation**: The drive and enthusiasm that young players must train, improve, and perform well in football, often fueled by their passion for the game, goals, and support from coaches and peers.

Based upon the previous discussion, this study will address the following research question: To what extent do the coach's feedback affect young football players’ experience of well-being, mastery, pleasure, satisfaction, development, motivation, and being seen by the coach? The hypothesis for this study is that feedback increases the experience of these factors among young football players, especially players that have chosen specialization in football at school.

## Method

To investigate the extent to which feedback during football training affects football players’ experiences of certain psychological variables, an intervention study was conducted. An experimental crossover design was used in which the players completed the same training session with and without feedback, and questionnaires were used after both training sessions.

Ethical approval was not required for the study involving humans in accordance with the local legislation and institutional requirements, but the study is in accordance with the guidelines for research ethics in the social sciences and the humanities (18). The study was approved by Norwegian data protection agency (SIKT, ref. code no 528649). All the players (or the parents if they were under the age of 16) that participated provided a written consent in accordance with regulations of SIKT.

### Participants

A list was made of all boys’ football teams within the league of G16 [the players are between 14 and 16 years old] in a large region in Norway that participated in sports clubs, as well as classes in sports studies with a specialization in football in the same city, as we hypothesized that the use of feedback would be perceived as more important for players in sports studies with a specialization in football. A random sample was used (19), and four boys’ teams and two sports studies classes with a specialization in football were randomly selected from these teams (see Table 1).

**Table 1:** Training session.

Participants	Number (*N*)	Age
G16 sports club participants	63	14–16
Sports studies specialization in football	32	16–18
All participants	95	14–18

### Development of a questionnaire

A questionnaire that measured perceived well-being, mastery, joy, satisfaction, development, and whether they wanted such sessions in the future, was developed based upon the definitions introduced in the introduction section. The questionnaire had a total of 11 questions—one measuring each variable, and each with a high face validity, that is highlighted as important (20). The questions were designed to measure the variables according to the definitions in the introduction section: Well-being was measured with the question: “I have enjoyed the football training as it has been today”. Mastery was measured with the question: The sense of competence and skill development in football, where young players feel they are improving and mastering new techniques and strategies. Pleasure was measured with the question: “I have experienced mastery in the football training as it has been today”. Satisfaction was measured with the question: “I have been satisfied with the football training as it has been today”. Development was measured with the question: “I feel I am developing as a football player with the football training as it has been today”. Being seen by the coach was measured with the question: “I felt seen by the coach during this training”. Motivation was measured with the question: “The training has been motivating today”. Furthermore, the question: “I feel that I received a lot of feedback from the coach during football training today” was included to examine if the players experienced the training with and without having feedback differently according to the rate of feedback. Finally, the questions “I want to have the football training as it has been today,”, “I do not want to have the football training as it has been today”, and “the training today has made me a better football player” were included to examine if the players preferred feedback or no feedback, and to include a different question related to development. The players were to respond based on a Likert scale from 1 to 5 (21), where 1 = strongly disagree, 2 = disagree, 3 = neither agree nor disagree, 4 = agree, 5 = strongly agree. Five-point Likert scales are commonly used in questionnaire studies, and the five answer options with one at each end, one in the middle (medium level), and the option between the middle and each end—are both valid and reliable in the case of inclusion. A study showed that data quality, internal consistency, and discriminative validity suggest that the five-point scale version should be used in research (22).

### Description of the intervention and data collection

The intervention consisted of all players participating in the same training session twice—one session with feedback and one session without feedback. A crossover design was used (19), where the three teams were randomly assigned to receive feedback either in the first or the second training session. An experimental crossover design is particularly relevant for this study for several reasons according to Thomas et al. (19). It controls individual differences, where each player experiences both conditions (with feedback and without feedback), so any observed differences in the outcomes can be more confidently attributed to the presence or absence of feedback. By having each participant serve as their own control, the design reduces variability and increases the statistical power of the study. This makes it easier to detect significant effects of feedback on the measured outcomes. The design allows for a direct comparison of the same players’ experiences under both conditions. This is more reliable than comparing different groups of players, as it eliminates between-group variability. Furthermore, a crossover design maximizes the use of available participants by having them experience both conditions, making the study more feasible and efficient. Finally, a crossover design ensures that all participants receive the potential benefits of feedback at some point during the study, which can be important for maintaining ethical standards and participant motivation. The first author was the coach for every training session. He has a UEFA C License, 4.5 years of sports education at a university, and one year of experience as a football coach. The second author was involved in the discussion of the intervention and the design. He has a UEFA C License, 9 years of sports education at a university, and 10 years of experience as a football coach the training session was organized as a typical and traditional football training for this age group based on the recommendations of the Norwegian Football Federation, and the same coach conducted all the training sessions. The exercises were developed based on traditional football training sessions. The training session was divided into three parts: warm-up (general and specific), main part, and conclusion (Table 2), and consisted of exercises that are central to the Norwegian Football Federation's development plan.

**Table 2:** Description of the training session.

Activity	Organization	Feedback^a^
Warm-up—injury prevention 10–15 min	Set up stations for injury prevention: - Run four laps - Inside movements - Knee lifts - Butt kicks - Hip swings - Indian hops - Groin in and out movements - Kick forward and back - Increased speed - Knee lifts, butt kicks, hip swings - Dog runs, forward two cones, back one - Stride runs 60%, 80%, 90%	Corrections on various exercises that can be done incorrectly Encouragement as a form of motivation Desire for better quality
Passing exercise 15–20 min Different variations: Turn up and move the ball to the side. Switch sides.	In this exercise, athletes are challenged with movement, orientation, and passing quality. 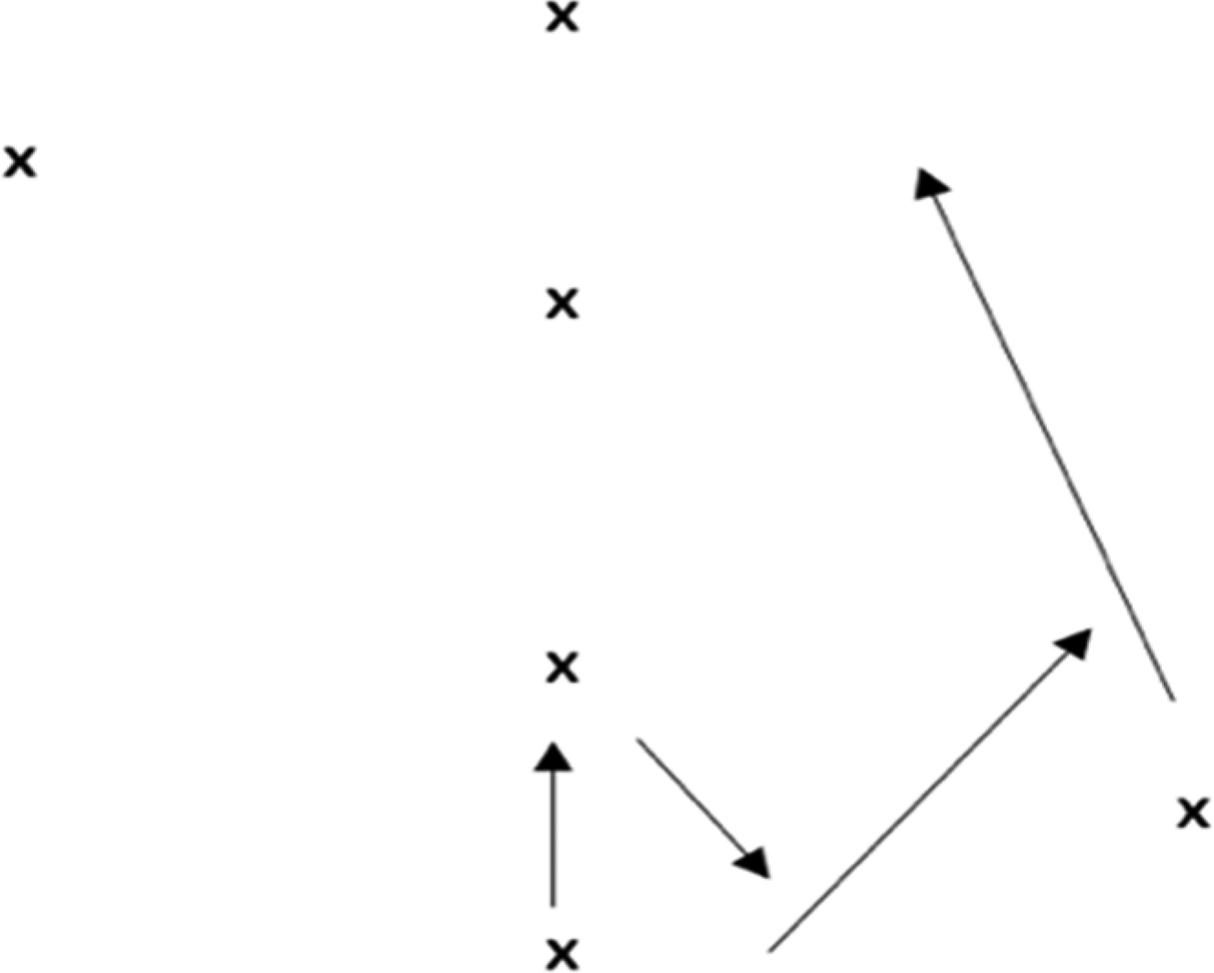	Passing quality, improvements Curved run Movement and countermovement Light on feet Talking/communication
Main part: possession—barca 20 min Play 30 min	Ball possession exercise focusing on orientation and switching sides. One from each team on each short side. Points for playing the ball over without the opponent touching it. Many of the same elements from the previous exercise are included in this one. Set up a confined rectangular area. < Focus upon aspects related to previous exercises	Passing quality, improvements Curved run Movement and countermovement Light on feet Talking/communication
Conclusion 5 min	Summary of the session Completion of the questionnaire	What was good and what to work more with

^a^ The feedback only took place in the training session with feedback, not in the session without feedback.

For the general warm-up, the injury-free exercise recommended by the Norwegian Football Federation was carried out. The main part consists of a possession exercise called “Barca,” including a game segment. Before the session, the organization of the training session and survey were described and explained to the participants without revealing the purpose of the study. Almost all sessions were conducted for 90 min, but in two of the sessions, it was “only” 60 min. This was the same group, so both training sessions had the same structure, organization, and time available.

*During the feedback sessions*, the coach was active in providing feedback by praising what was good and guiding on what could be improved. Some players took this feedback positively, while others did not. This may be because they were not entirely comfortable in the situation, perhaps because they did not feel secure with the new coach. During the first passing exercise, it was easier to give feedback because the coach could stand in one place and observe all the participants. In the possession exercise, it was more challenging to give feedback to everyone. When situations related to what was being practiced arose, the coach stopped the game to show and explain the situation. The players were also asked what they thought they did well or could have done better during the training. Sometimes the game was “frozen” in the game situation to either praise positive things we had practiced or to show the participants various situations where there was potential for improvement. A similar approach was used during the game itself. *In the sessions without feedback*, no individual feedback was given. Only messages were given in plenary before or after the exercise, or during game stops to give messages regarding the organization of the training.

The data collection took place between January–April 2024. Both training sessions (both with and without feedback) were conducted on the artificial turf fields of the respective teams, at the normal training times for the teams. In this way, the two training sessions took place at the same location and at the same time. After each training, the players were given a questionnaire that measured perceived well-being, mastery, joy, satisfaction, development, and whether they wanted such sessions in the future, based on what they had experienced in the session.

The experimental design and implementation had some potential biases or limitations. First, the participants’ perceived feeling of well-being, mastery, joy, satisfaction, and development may also depend on how they felt that day (the daily condition)—even if this would probably be randomly divided into days with and without feedback. Secondly, the coach's ability to give feedback during the feedback sessions, and not to give feedback during the sessions without feedback, is essential. Even if this is minimized by using the same coach in all sessions, and the results show that they experience getting feedback during the feedback training, this is a potential bias. Finally, the willingness to take the time to complete the questionnaire truthfully as best as possible after completing the training session is a potential bias.

### Statistical analysis

The data were plotted into SPSS with values from 1 to 5, using the Likert scale values from 1 to 5 and analyzed using IBM SPSS Version 29. Checking the data for normal distribution showed that the criteria of normal distribution related to parametric tests were not met (Kolmogorov–Smirnov = *p* < 0.05), and the variables were not at an interval or ratio level. According to the assumptions of parametric tests, the Wilcoxon non-parametric test was used to identify differences between the training sessions with or without using feedback (21, 23). Significant differences were set at *p* ≤ 0.05, and all data are presented as mean ± standard deviation.

## Results

**Figure 1:**
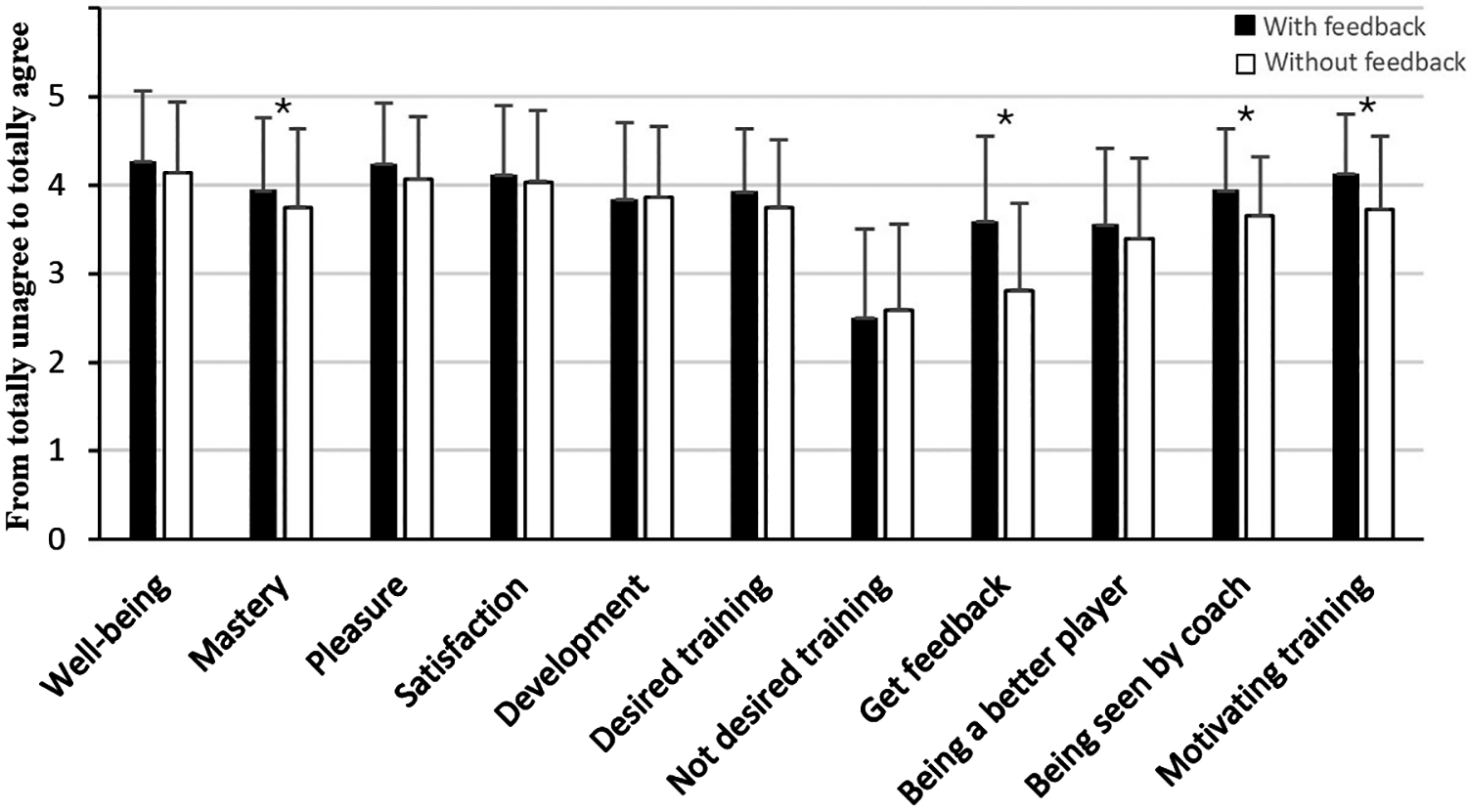
Mean and standard deviations of psychological variables in football training with and without getting feedback. *Significant difference between training with and without getting feedback at a 0.05 level.

The statistical analyses of all players showed that when feedback was given, it led to a significant increase in mastery (Z = −2.2, *p* = 0.029), the experience of being seen by the coach (Z = −3.3, *p* = 0.001), and motivation (Z = −3.8, *p* = 0.001), compared to the same training session when they did not receive feedback (Figure 1). The players also significantly experienced receiving feedback to a greater extent in the training session where feedback was given, compared to the same training session when they did not receive feedback (Z = −5.5, *p* = 0.001). However, according to experienced well-being, pleasure, satisfaction, development, desired or not desired training, and being a better player, increased feedback did not have an impact on those variables (*p* > 0.05).

**Figure 2:**
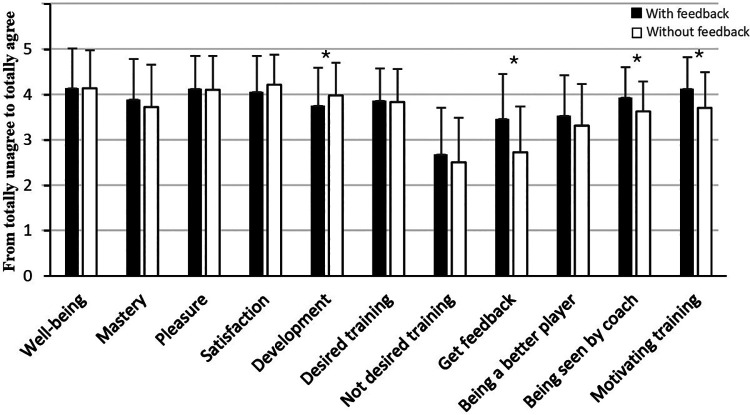
Mean and standard deviations of psychological variables in football training with and without getting feedback among players who participated in sports teams and did not specialize in football in sports programs. *Significant difference between training with and without getting feedback at a 0.05 level.

The statistical analyses of the players who participated in sports teams and did not specialize in football in sports programs showed that when feedback was given, it led to a significant increase in development (Z = −2, *p* = 0.050), the experience of being seen by the coach (Z = −2.8, *p* = 0.005), and motivation (Z = −3.4, *p* = <0.001), compared to the same training session when they did not receive feedback (Figure 2). The players also significantly experienced receiving feedback to a greater extent in the training session where feedback was given, compared to the training session where they did not receive feedback (Z = −4.6, *p* = <0.001). However, according to experienced well-being, mastery, pleasure, satisfaction, desired or not desired training, and being a better player, increased feedback did not have an impact on those variables (*p* > 0.05).

The statistical analyses of players that participated in sports studies with a specialization in football showed that when feedback was given, it led to a significant increase in well-being (Z = −2.7, *p* = 0.007), pleasure (Z = −2.7, *p* = 0.006), satisfaction (Z = −2.8, *p* = 0.005), and development (Z = −2.1, *p* = 0.039), compared to the same training session when they did not receive feedback (Figure 1). The players also significantly experienced receiving feedback to a greater extent in the training session where feedback was given, compared to the training session where they did not receive feedback (Z = −3.1, *p* = 0.002). Additionally, they desired feedback through both positive and negatively framed questions (respectively Z = −3.2, *p* = 0.002 and Z = −2.8, *p* = 0.005). However, according to experienced mastery, being seen by the coach, being a better player and motivation, increased feedback did not have an impact on those variables (*p* > 0.05).

## Discussion

The aim of the study was to examine how the coach's feedback affected psychological variables among young football players’ experiences of well-being, mastery, pleasure, satisfaction, development, motivation, and being seen by their coach. The results show that the coach giving feedback has a positive impact on several psychological variables among young football players, but this effect varies depending on different player groups. At the same time, the players report that they received significantly more feedback in the training session where feedback was given, which helps confirm the validity of the experimental design on which the study is based. The results of the study confirmed the hypothesis, showing a significant increase in the levels of the examined psychological variables following the implementation of feedback. Our findings are supported by the findings of Weakley et al. (13), who found that enhanced psychological variables such as motivation and perceived effort were affected positively by feedback. In their study physiological variables such as muscular endurance, speed, strength and jump performance demonstrated a positive influence of feedback, with all outcomes showing superior results compared to when no feedback was provided.

We argue that the positive effect of feedback can be attributed to several factors. Previous research has highlighted the importance of feedback as a central factor in young people's experience of being seen in physical education (4, 5). Our findings in the present study show that the experience of being noticed is significantly higher in sessions with feedback when all players are analyzed together. Author a found that of those who were ’seen’, most received a great deal of feedback from their PE teachers, whereas those who were not ’seen’ felt that they received very little or no feedback.

According to Author a, recognition can be related to feeling seen. Jordet's (6) recognition theory points to the experience of being seen, being listened to, and showing attention as fundamental to creating social relationships. That participants feel more recognized when they receive feedback may indicate that feedback is an important tool in meeting participants’ needs to be seen (6). Jordet's recognition theory supports the positive effect of feedback. In this theory, love’ refers to the importance of showing understanding and care, which can create a bond between student and teacher. This can increase well-being and joy, and here the use of feedback can be important. Another factor that can affect well-being is that the teacher gives feedback that is personal and appropriate for the students. Also, Lyngstad et al. (24) found that when the PE teacher gave feedback, the students reported being seen by their PE teacher. Furthermore, the importance of feedback is closely related to Federici and Skaalvik's (25) description of emotional and instrumental support, which is about giving instrumental support when the teachers give advice, support and guidance, and the emotional aspect involving encouragement, appreciation, and a teacher who cares.

According to our findings related to a higher experience of being seen by the coach when feedback was given, Cox et al. (26) highlight that adolescents who feel supported by their teacher also have a greater sense of belonging, and belonging is described by Deci and Ryan (7) as a fundamental need, which will therefore affect the experience of being seen. With feedback, the players feel their competencies are valued and recognized (6), and that the coach feedback is a strategy that involves giving praise and appreciation, and acknowledgment of what the person does. Hattie and Timperley (1) also highlight the positive effect of feedback on development and performance improvement. According to Hattie and Timperley, feedback is particularly important for development, and coaches promote development through daily immediate feedback provided during the development process. The questions are “where is the player, where should the player go, and how does the player get there?” Through feedback, football coaches can evaluate athletes’ performances against expectations and provide instructions and guidance on how they can achieve and improve these expectations.

The findings of the study showed that when feedback was given, it led to a significant increase in mastery when all players were included, but also an increase in motivation. This finding is supported by Gökçe's (11) study, which found that positive feedback helped increase students’ mastery and goal orientation. One can argue that this underscores the importance of feedback and its role in promoting motivation, mastery, and development.

Social recognition in recognition theory shows that motivation and mastery are important contributors to feeling recognized (6), and here feedback is important. Players who feel valued in a social setting through being recognized via feedback will have a higher degree of effort, increased interest in work, and a more positive attitude towards development as football players according to Jordet's recognition pedagogy. The findings of increased mastery and motivation when feedback was given can also be explained by self-determination theory (7), where feedback increases the players feeling of competence and social belonging—which are fundamental needs.

In Smith and St. Pierre's (17) study, most students claimed that the teacher had a significant impact on their experience of physical education, and that students’ well-being was greatly influenced by the interaction between teacher and student. The teacher's ability to communicate effectively with students, as well as encouragement and providing positive feedback, was an important factor for well-being in this study. We believe that this also applies to coaches on a football field.

Lyngstad et al.'s (24) study points out that good communication through feedback between teacher and student (here, coach and player) can affect the experience of being seen. Therefore, it is appropriate to provide clear feedback that is tailored to the individual.

Based on the study's findings related to the positive effect of feedback, an important question is how to provide feedback. Otte et al. (27) point out six guidelines aimed at supporting sports coaches in providing feedback. The first is that the training design that facilitates athletes’ self-regulation in sport should always be at the core of all learning and coaching activities and is important to provide highly valuable intrinsic feedback. The second is the coach's understanding of the athlete's particular skill development and training stage, which is paramount for appropriate selection of feedback and instruction methods. This stands in contrast to the Performance Training stage, which due to immediate performance and time pressure may require coaches to apply a more targeted and direct communication style. The third guideline is that in contrast to the common notion, “the more, the better”, athletes at skill developmental stages benefit more from self-regulatory approaches and minimized explicit feedback and instructions used sparingly. The fourth guideline is that the timing of visual feedback is also important for athletes to perceive and use intrinsic information from movements to self-regulate in solving ongoing performance problems. Coaches should delay the provision of augmented feedback to provide time for athletes to perceive movement feedback for use in ensuing practice tasks. The fifth guideline is that augmented verbal information should avoid a specification of precisely how an athlete should solve a performance problem. The wording of feedback and instructions should be used to stimulate and elicit further exploration of specific opportunities for action. Consequently, the coach should act as a “moderator” to guide athletes’ search and problem-solving for functional (movement) solutions. The sixth and last guideline is that the feedback and instruction methods that athletes seek and the way that individual athletes respond to these should drive coaches’ communication. In this respect, an understanding of athlete-centered coaching is necessary.

**Figure 3:**
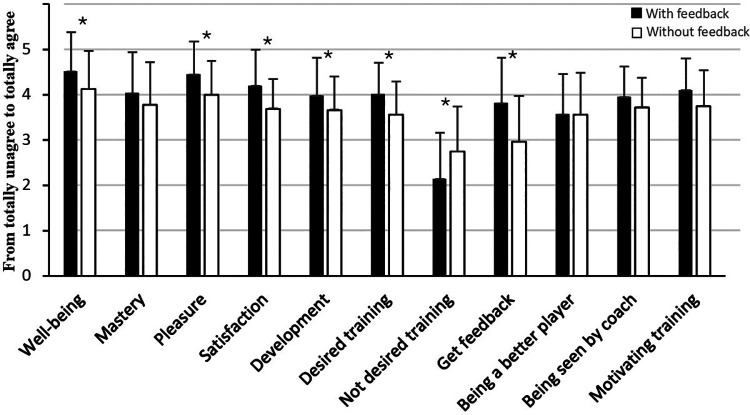
Mean and standard deviations of psychological variables in football training with and without getting feedback among players who specialize in football in sports programs. *Significant difference between training with and without getting feedback at a 0.05 level.

Another main finding of the study was that feedback had the most positive effects on players that participated in sports studies with a specialization in football, where feedback led to a significant increase in both well-being, pleasure, satisfaction, and development. Furthermore, an unexpected result was the experience of development within training sessions with or without feedback, where players who participated in sports studies with a specialization in football increased their experience of development with feedback in contrast to players who participated in sports teams and did not specialize in football in sports programs, where feedback decreased their feeling of development. A possible explanation for these two findings may be that players with a specialization in football have chosen to have this specialization to develop as players, and that development is of more importance to them. Previous research has shown that getting feedback is important for development (1, 2, 9). Players who participated in sports teams and did not specialize in football in sports programs, may be more into football for social reasons than for development (28). This explanation is supported by the results related to the desired and not desired training in Figures 2, 3, where players who participated in sports studies with a specialization in football significantly desired training with feedback more than training without feedback, while players who participated in sports teams and did not specialize in football in sports programs did not significantly prefer training with feedback more than training without feedback. That athletes who did not specialize in football in sports programs felt greater development in sessions without feedback, suggests that autonomy and self-reflection may play an important role in the experience of progress. This may be because athletes feel greater ownership of their development when they do not receive feedback.

### Limitations and future research

The use of an experimental crossover design where the players completed the same training session with and without feedback with the same coach and under the same conditions, fulfilling the same questionnaire immediately after each training session is a strength of the study. However, the questionnaire was not based upon a pre-validated instrument, because such a questionnaire does not exist. Although the questions and the reply options have a high face validity (20), which should not lead to different interpretations of the questions, and we used validated Likert scales (22). We argue that both the questions and the answer options contributed to high reliability. Furthermore, the players reported that they received significantly more feedback during the training session where feedback was given, which helps to confirm the validity of the study. Finally, as highlighted in the methods section the daily condition of the players, the coach's ability to give feedback during the feedback sessions, and not to give feedback during the sessions without feedback, and the players’ willingness to take the time to complete the questionnaire truthfully are potential biases.

## Conclusion

To the best of our knowledge, this is the first study to examine the impact of feedback on psychological factors of young football players, using an experimental intervention study. The findings of this study show that sessions with feedback have a positive impact on several psychological factors of young football players, such as mastery, motivation, and the experience of being seen by their coach. The practical application of the study is that feedback should be used as a tool to promote a more engaged and positive environment in football training by providing highly valuable intrinsic feedback, especially among elite athletes. It turns out that in the sessions with feedback, 80% of the participants reported feeling noticed. The results showed that especially elite athletes preferred sessions with feedback and reported increased well-being, pleasure, satisfaction, and development when receiving feedback from their coaches. The positive impact of feedback is supported by previous research by (1, 2), which emphasizes the importance of feedback and how it promotes development. The practical implication of the study should also be that football coaching courses should highlight the importance of giving feedback, and teach their participants how to provide feedback to young players. Future studies should focus on what makes athletes and students experience feedback and how they best perceive feedback, using a qualitative approach. This will help expand the knowledge base around feedback and how to provide students and players with more precise and effective use of feedback as a tool for skill development in football. It is also recommended to include interventions that take place over a longer period. According to Hattie and Timperley (1), feedback that is not personalized can lead to misunderstandings or reduce quality, and it is recommended that future studies have interventions over a longer period to improve the effectiveness of feedback and development. This will make coaches more familiar with the participants and thus enable them to provide more concrete and relevant feedback. Future research should address the limitations in this study by examining the effects of feedback using pre-validated instruments and including more coaches and players in the study.

## Data Availability

The raw data supporting the conclusions of this article will be made available by the authors, without undue reservation.
